# Author Correction: Two point mutations in protocadherin-1 disrupt hantavirus recognition and afford protection against lethal infection

**DOI:** 10.1038/s41467-023-41659-y

**Published:** 2023-09-22

**Authors:** Megan M. Slough, Rong Li, Andrew S. Herbert, Gorka Lasso, Ana I. Kuehne, Stephanie R. Monticelli, Russell R. Bakken, Yanan Liu, Agnidipta Ghosh, Alicia M. Moreau, Xiankun Zeng, Félix A. Rey, Pablo Guardado-Calvo, Steven C. Almo, John M. Dye, Rohit K. Jangra, Zhongde Wang, Kartik Chandran

**Affiliations:** 1 Department of Microbiology and Immunology, Albert Einstein College of Medicine, Bronx, NY USA; 2 Department of Animal, Dairy and Veterinary Sciences, Utah State University, Logan, UT USA; 3 United States Army Medical Research Institute of Infectious Diseases, Fort Detrick, MD USA; 4 The Geneva Foundation, Tacoma, WA USA; 5 Department of Biochemistry, Albert Einstein College of Medicine, Bronx, NY USA; 6 Institut Pasteur, Université Paris Cité, CNRS UMR3569, Structural Virology Unit, F-75015 Paris, France; 7 Institut Pasteur, Université Paris Cité, Structural Biology of Infectious Diseases Unit, F-75015 Paris, France; 8 Present Address: Microbiology and Immunology, Louisiana State University Health Sciences Center-Shreveport, Shreveport, LA USA

**Keywords:** Virus-host interactions, Viral reservoirs, Hamster

Correction to: *Nature Communications* 10.1038/s41467-023-40126-y, published online 24 July 2023

The original version of this article contained an error where Rohit K. Jangra and Zhongde Wang were not listed as corresponding authors and their email addresses rohit.jangra@lsuhs.edu and zonda.wang@usu.edu were not included. This has been corrected in both the PDF and HTML versions of the Article.

