# Supplementary material for: Two point mutations in protocadherin-1 disrupt hantavirus recognition and afford protection against lethal infection
Source: Nat Commun. 2023 Jul 24;14:4454. doi: 10.1038/s41467-023-40126-y (PMC10366084; doi:10.1038/s41467-023-40126-y)
Supplement: Supplementary file 1 — Supplementary information [file 41467_2023_40126_MOESM1_ESM.pdf]

Two point mutations in protocadherin-1 disrupt hantavirus recognition and afford protection against lethal infection

Megan M. Slough, Rong Li, Andrew S. Herbert, Gorka Lasso, Ana I. Kuehne, Stephanie R. Monticelli, Russell R. Bakken, Yanan Liu, Agnidipta Ghosh, Alicia M. Moreau, Xiankun Zeng, Félix A. Rey, Pablo Guardado-Calvo, Steven C. Almo, John M. Dye, Rohit K. Jangra, Zhongde Wang, Kartik Chandran

Supplementary Information

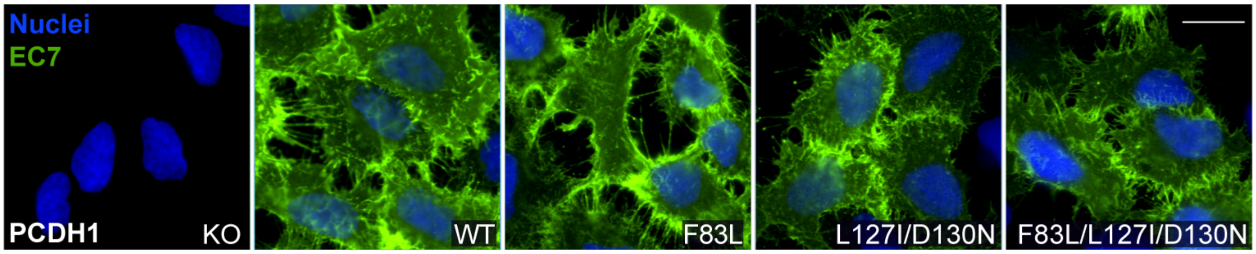

Supplementary Figure 1. Overexpression of human PCDH1 bearing mouse mutations exhibit similar expression levels to wild-type PCDH1

Surface expression of PCDH1 variants. U2OS *PCDH1*-KO cells, expressing the indicated PCDH1 variants, were immunostained with an anti-PCDH1, EC7-specific “3677” monoclonal antibody. Representative images from one experiment are shown. Scale bar, 20 μm.

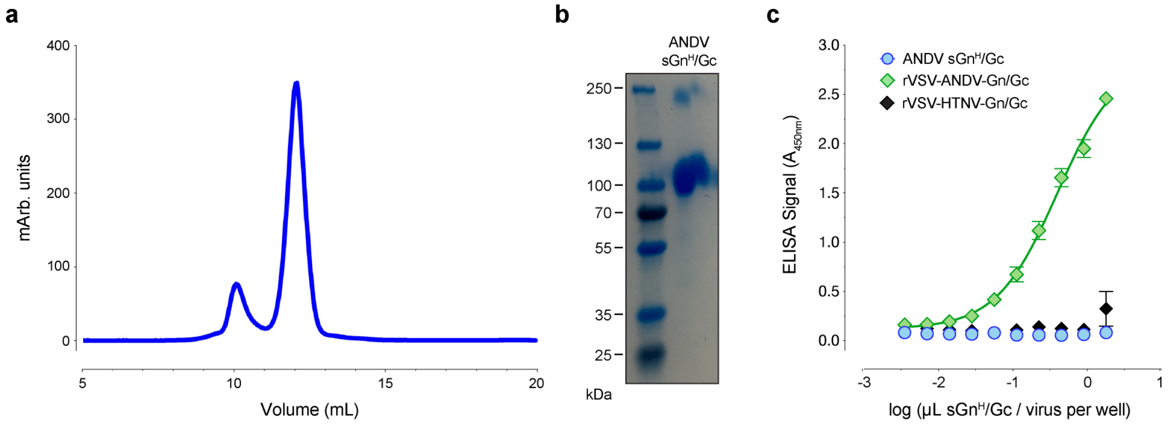

Supplementary Figure 2. Soluble ANDV Gn^{H}/Gc does not bind to soluble EC1-2

(a) Size exclusion chromatography profile of soluble ANDV Gn^{H}/Gc protein (sGn^{H}/Gc). The protein elutes in two peaks that correspond to an oligomeric and a monomeric form. Binding experiments to sEC1-2 were performed with the monomeric fraction (the second, taller peak) (mArb., milli arbitrary units). (b) To confirm the sGn^{H}/Gc had not undergone degradation, concentrated protein was separated on an SDS-polyacrylamide gel and visualized by Coomassie Brilliant Blue staining. The expected molecular weight of sGn^{H}/Gc is around 106 kilodalton (kDa) (97 kDa for the protein, plus nine kDa for the three glycosylation sites). A molecular weight ladder is the first lane. A representative image from one of two independent experiments is shown. (c) ELISA detection of bound sGn^{H}/Gc and rVSVs bearing ANDV or HTNV Gn/Gc. ANDV sGn^{H}/Gc was diluted to an initial concentration of 3.5 µg/µL (6.3 µg or 1.8 µL protein/well) and serial diluted twofold. Normalized FSL-biotin labeled rVSVs expressing ANDV or HTNV Gn/Gc were serial diluted twofold (initial dilution 1.8 µL virus/well). Diluted ANDV sGn^{H}/Gc and viral particles were added to sEC1-2(WT) coated ELISA plates. Bound ANDV sGn^{H}/Gc was detected using a Strep-Tactin®-horseradish peroxidase (HRP) conjugate and bound viral particles were detected using a Pierce™ High Sensitivity Streptavidin-HRP conjugate. Means ± SEM: n = 6 wells per dilution of ANDV sGn^{H}/Gc and rVSV-ANDV-Gn/Gc examined over two independent experiments, n = 3 wells per dilution of rVSV-HTNV-Gn/Gc examined over one experiment. (FSL: functional-component spacer diacyl lipid, sEC1-2: soluble extracellular cadherin domains 1 and 2). Source data are provided as a Source Data file.

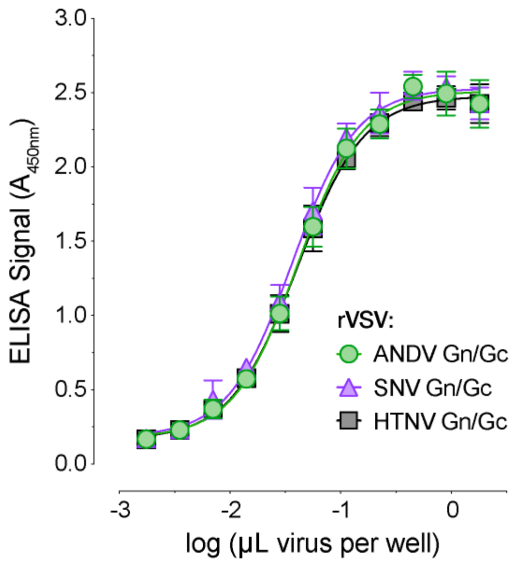

Supplementary Figure 3. Quantification of rVSV particles expressing hantavirus Gn/Gc
ELISA comparing normalized rVSV particles. FSL-biotin labeled rVSVs expressing ANDV, SNV, or HTNV Gn/Gc were serial diluted twofold and coated onto ELISA plates. Viral particles were detected using a Pierce™ High Sensitivity Streptavidin-HRP conjugate. Means ± SD: n = 6 wells per dilution of virus examined over three independent experiments (n = 5 wells for four dilutions of ANDV and n = 5 wells for one dilution of SNV and HTNV). (FSL: functional-component spacer diacyl lipid). Source data are provided as a Source Data file.

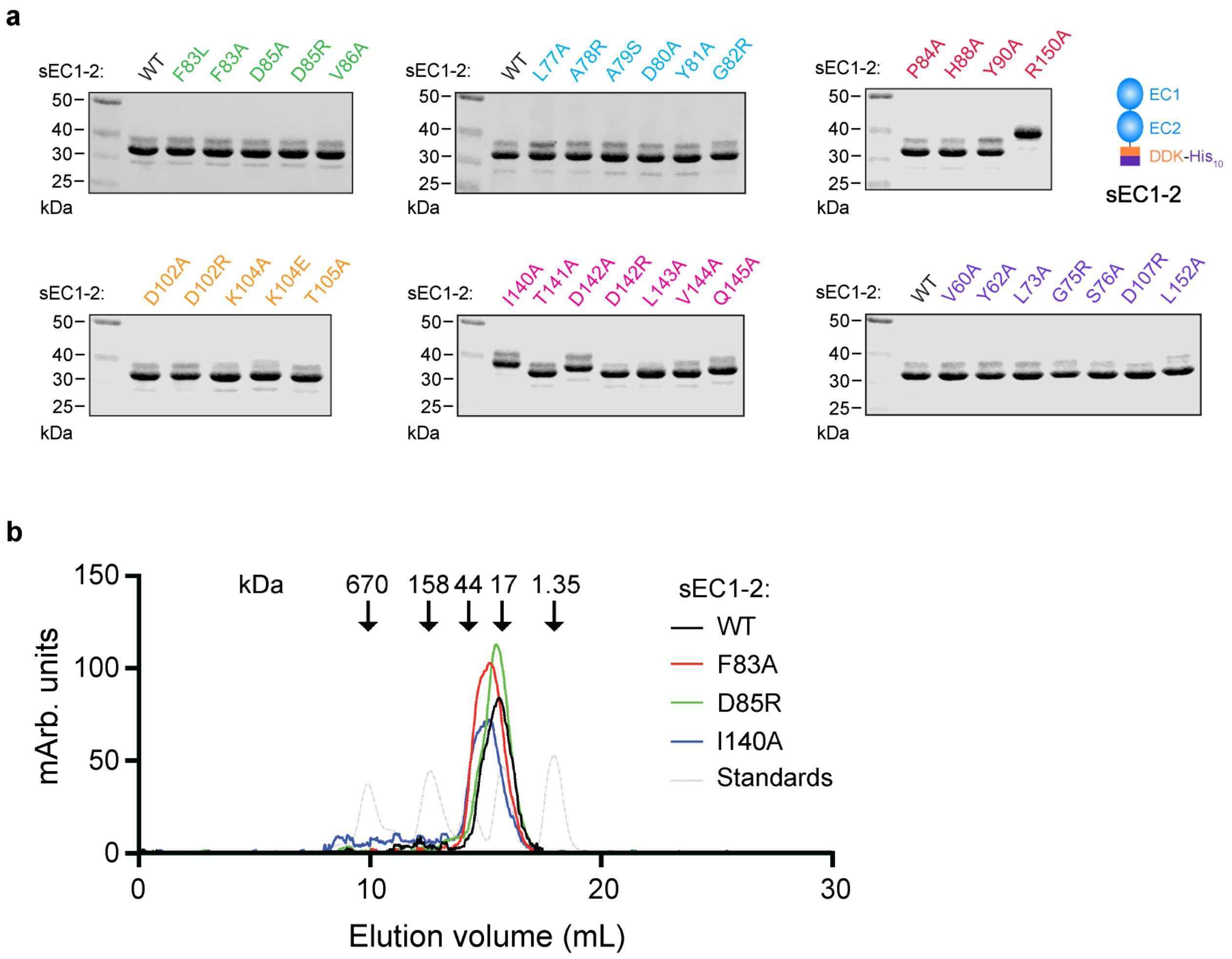

Supplementary Figure 4. Expression and purification of PCDH1 soluble EC1-2 mutants
(a) Purified WT and mutant sEC1-2 proteins were separated on an SDS-polyacrylamide gel and visualized by Coomassie Brilliant Blue staining. The molecular weight ladder is in the first lane of each gel (kDa, kilodalton). Schematic representation of sEC1-2 (right). A single experiment was performed for each protein except for WT sEC1-2, which was examined over twelve independent experiments. (b) The elution profile of size exclusion chromatography (SEC) of WT and a selection of mutant sEC1-2 proteins from (a). Absorbance (mArb, milli arbitrary units) was measured at 280 nm using a calibrated Superdex S200 column at a physiological salt concentration. The dotted gray line shows elution profiles of SEC standards (molecular weights indicated) under the same buffer conditions. (sEC1-2: soluble extracellular cadherin domains 1 and 2).

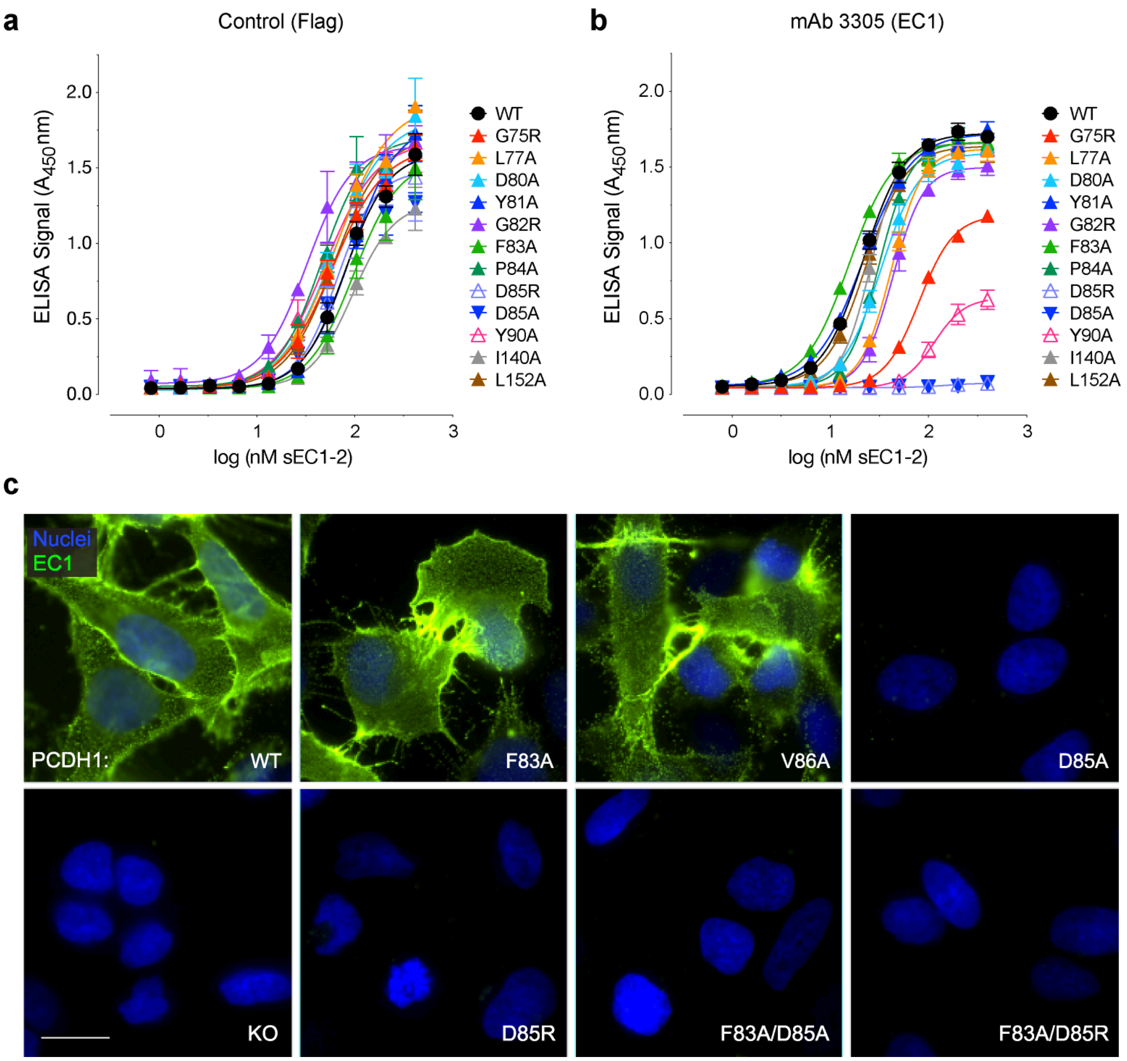

Supplementary Figure 5. New World hantavirus infection-blocking 3305-mAb recognizes PCDH1 EC1 residue D85

(a) ELISA detecting Flag-tagged WT and mutant sEC1-2 proteins. sEC1-2 variants were serially diluted twofold and coated onto ELISA plates. Protein was detected using an anti-Flag-HRP antibody. Averages ± SD: n = 4 wells of each dilution of protein examined over two independent experiments [n = 3 wells for one dilution for sEC1-2(G82R)]. (b) The capacity of anti-EC1 3305-mAb to bind to WT or mutant sEC1-2 proteins. Averages ± SD: n = 4 wells for each dilution of protein examined over two independent experiments. Done in parallel with (a). (c) Detection of PCDH1 variants using anti-EC1 3305-mAb on U2OS *PCDH1*-KO cells complemented with WT or mutant PCDH1. Although the cells expressing the PCDH1(D85) variants are not stained here, the protein is expressed in these cells (Supplementary Figure 6) using an EC7-specific, 3677-mAb. Representative images from one experiment of two independent experiments are shown. Scale bar, 20 µm. Source data are provided as a Source Data file.

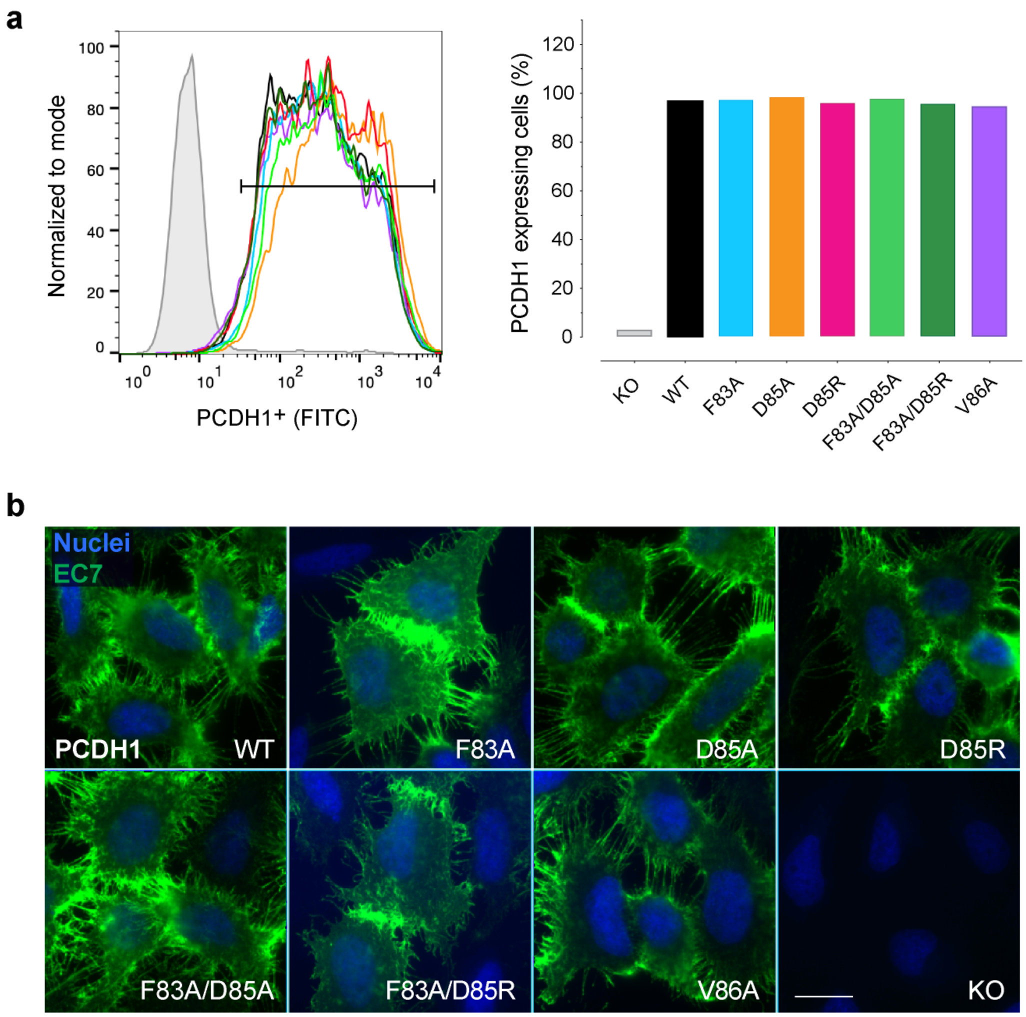

Supplementary Figure 6. Overexpression of mutant PCDH1 exhibit similar expression levels to WT

(a) Flow cytometry histogram of PCDH1 expression and percent PCDH1^{+} cells (U2OS *PCDH1*-KO cells complemented with WT or mutant PCDH1). The cell surface-expressed PCDH1 protein was detected by an EC7-specific 3677-mAb and analyzed using flow cytometry. Data from one representative experiment is shown. (b) Surface expression of PCDH1 on cell lines from (a) visualized using an EC7-specific 3677-mAb. Scale bar, 20 µm. Representative images from a single experiment of two independent experiments are shown.

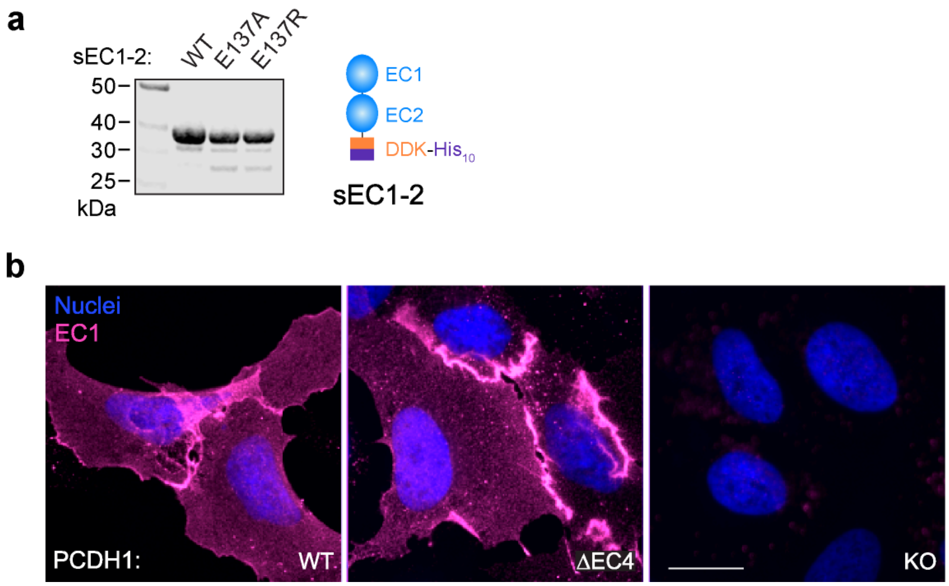

Supplementary Figure 7. Mutations disrupting the EC1:EC4 interface in soluble PCDH1 and monomeric PCDH1 cell lines exhibit similar expression levels to WT
(a) Purified WT and mutant sEC1-2 were separated on an SDS-polyacrylamide gel and visualized by Coomassie Brilliant Blue staining. kDa, kilodalton. A single experiment was performed to visualize the mutant soluble proteins. Schematic representation of sEC1-2 (right). (b) Cell surface expression of PCDH1 on U2OS *PCDH1*-KO cells complemented with WT or mutant-without the EC4 domain (ΔEC4) PCDH1 proteins. PCDH1 was immunostained using a PCDH1 EC1-specific 3305-mAb. Scale bar, 20 µm. Representative images from a single experiment illustrating two independent experiments are shown.

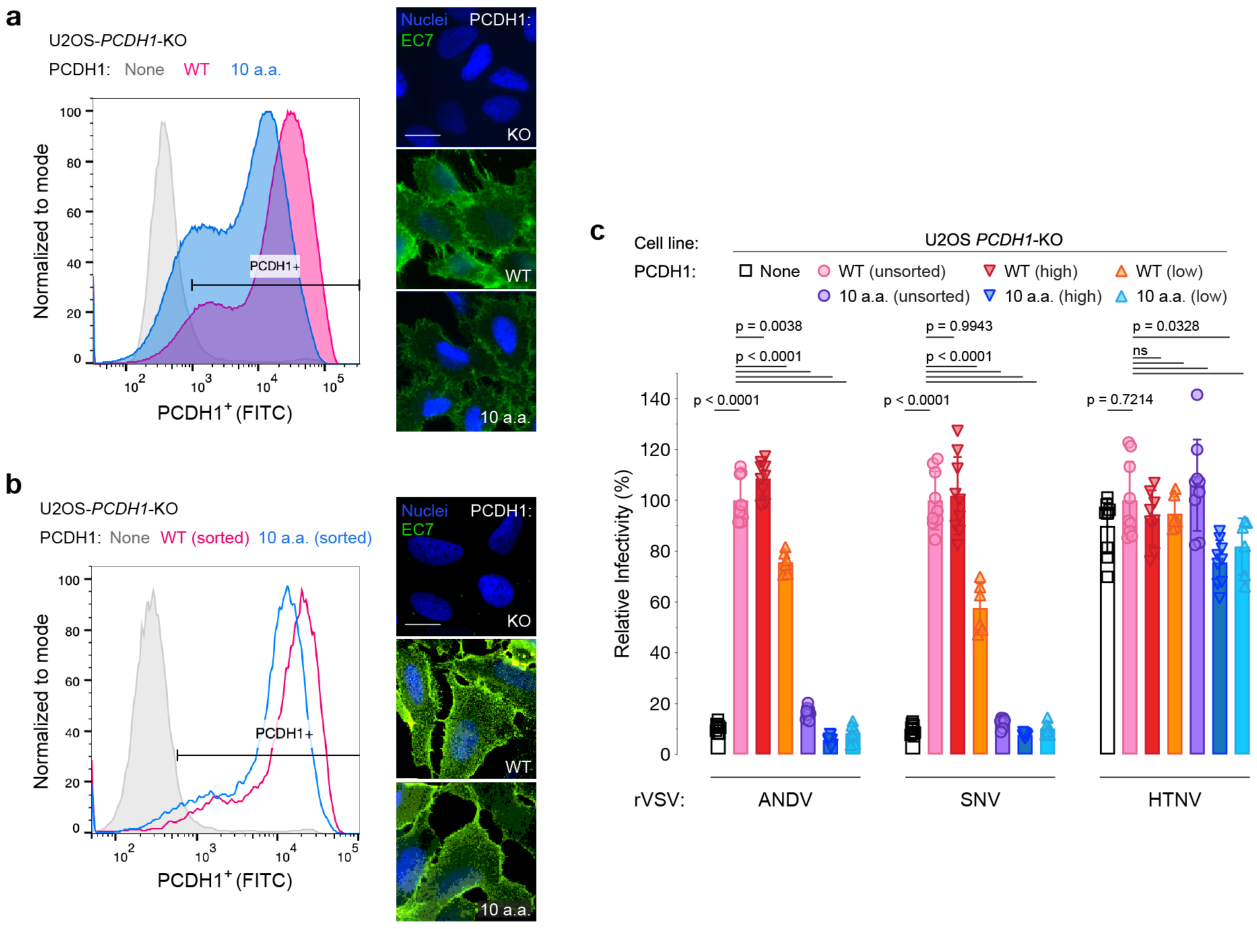

Supplementary Figure 8. Cells expressing high levels of PCDH1(10a.a.) mutant do not support ANDV Gn/Gc-mediated infection

(a) Flow cytometry histogram and immunofluorescence of cell surface PCDH1 expression on U2OS *PCDH1*-KO cells complemented with either WT or mutant PCDH1. The surface of the cells was immunostained using a PCDH1 EC7-specific 3677-mAb followed by analysis using flow cytometry or imaged via an inverted fluorescence microscope. The percent of $PCDH1^{+}$ cells are as follows; none (10.4%), low (81.8%), and high (93.0%). Representative flow cytometry histogram and representative fluorescent images, from two independent experiments, are shown. Scale bar, 20 µm. (b) Flow cytometry histogram and immunofluorescence images of sorted cells stained as described in (a). Cells described in (a) were sorted into high or low PCDH1 expression. Cells expressing high levels of PCDH1 are represented in the histogram and fluorescent images. The percent of $PCDH1^{+}$ cells are as follows; none (10.1%), low (94.0%), and high (95.7%). Flow cytometry histogram from one experiment is shown and representative fluorescent images from one experiment of two independent experiments are shown. Scale bar, 20 µm. (c) Relative infectivity of rVSVs bearing ANDV, SNV, or HTNV Gn/Gc on unsorted and sorted U2OS *PCDH1*-KO cells complemented with either WT or mutant PCDH1 described in (a) and (b). The infectivity of each virus was normalized to that obtained in U2OS *PCDH1*-KO cells complemented with WT PCDH1. Means ± SD: *n* = 9 wells of infected cells examined over three independent experiments (*n* = 8 wells for rVSV-HTNV-Gn/Gc infection on sorted WT-high PCDH1 expressing cells). For all sorted low expressing PCDH1 cell lines, *n* = 6 wells of infected cells examined over two independent experiments. Infectivities were compared by one-way ANOVA with Dunnett's test for multiple comparisons; *ns* > 0.05. Source data are provided as a Source Data file.

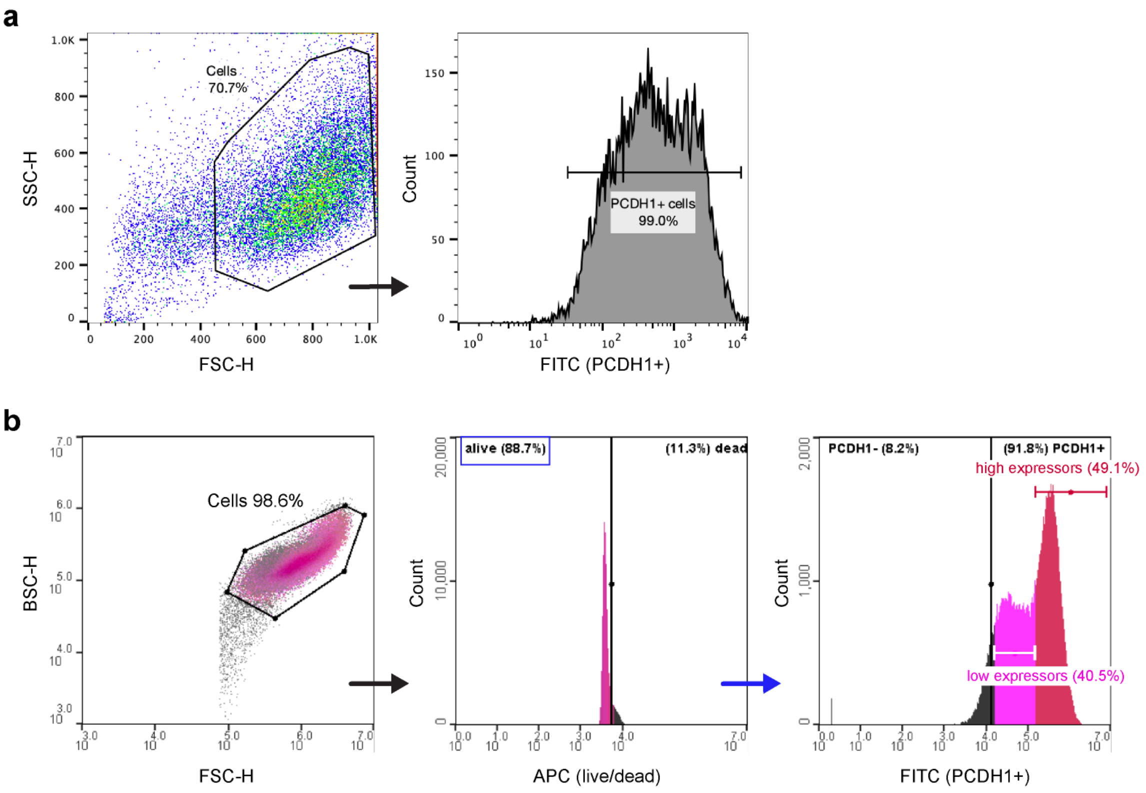

Supplementary Figure 9. Gating strategy for flow cytometry

(a) Gating strategy used for Supplementary Figure 6a displaying density plots generated from LSRII Flow Cytometer and analyzed by FloJo V.10 software. The first plot isolates cells from background debris by size: side-scatter height (SSC-H) versus forward-scatter height (FSC-H). K denotes 1,000. The second plot displays the isolated cell population from plot-1 as a histogram and includes a gate to identify cells that express PCDH1 on their surface (PCDH1+). The gate cutoff was determined using the signal from *PCDH1*-KO cells as a baseline. (b) Gating strategy used for Supplementary Figure 8a-b displaying density plots created by the NanoCellect, WOLF Cell Sorter. The first plot isolates cells from background debris by complexity (Back-scatter height, BSC-H) versus cell size (FSC-H); the second plot displays the cell population isolated in plot-1 as a histogram and gates for live cells (those that stained negative for TO-PRO™3 Ready Flow™ live-dead stain); the third plot displays the gated live cells in plot-2 as a histogram and includes gates to isolate and sort cells into three groups: non-PCDH1 expressing cells (PCDH1-) and high or low expressing PCDH1+ cells (high expressors and low expressors). The gate cutoff was determined using the signal from *PCDH1*-KO cells as a baseline.

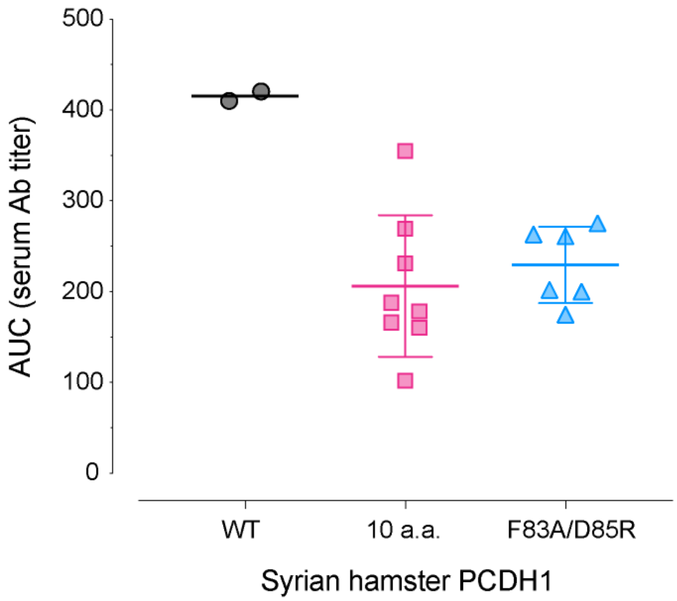

Supplementary Figure 10. Seroconversion of Syrian hamsters challenged with ANDV
Area under the curve (AUC) for ANDV Gn/Gc-specific antibodies from WT, PCDH1(10a.a.), and PCDH1(F83A/D85R) CRISPR knock-in mutant Syrian hamsters' sera. Syrian hamsters were challenged with 2,000 PFU (PFU, plaque forming units) of ANDV and serum titers were evaluated for all hamsters who survived until day 35. ANDV Gn/Gc-specific IgGs were detected using rVSV-ANDV-Gn/Gc coated ELISA plates. Each data point represents the average AUC values of single hamster examined over three independent binding experiments. Averages ± SD; WT: n = 2 hamsters, PCDH1(10 a.a.): n = 8 hamsters, and PCDH1(F83A/D85R): n = 6 hamsters. Source data are provided as a Source Data file.
