# Supplementary material for: Two point mutations in protocadherin-1 disrupt hantavirus recognition and afford protection against lethal infection
Source: Nat Commun. 2023 Jul 24;14:4454. doi: 10.1038/s41467-023-40126-y (PMC10366084; doi:10.1038/s41467-023-40126-y)
Supplement: Supplementary file 3 — Reporting Summary [file 41467_2023_40126_MOESM3_ESM.pdf]

nature portfolio

Corresponding author(s): Chandran, Wang, and Jangra

Last updated by author(s): Jun 13, 2023

Reporting Summary

Nature Portfolio wishes to improve the reproducibility of the work that we publish. This form provides structure for consistency and transparency in reporting. For further information on Nature Portfolio policies, see our Editorial Policies and the Editorial Policy Checklist.

Statistics

For all statistical analyses, confirm that the following items are present in the figure legend, table legend, main text, or Methods section.

n/a	Confirmed	
☐	☒	The exact sample size (*n*) for each experimental group/condition, given as a discrete number and unit of measurement
☐	☒	A statement on whether measurements were taken from distinct samples or whether the same sample was measured repeatedly
☐	☒	The statistical test(s) used AND whether they are one- or two-sided<br>*Only common tests should be described solely by name; describe more complex techniques in the Methods section.*
☒	☐	A description of all covariates tested
☐	☒	A description of any assumptions or corrections, such as tests of normality and adjustment for multiple comparisons
☐	☒	A full description of the statistical parameters including central tendency (e.g. means) or other basic estimates (e.g. regression coefficient) AND variation (e.g. standard deviation) or associated estimates of uncertainty (e.g. confidence intervals)
☐	☒	For null hypothesis testing, the test statistic (e.g. *F*, *t*, *r*) with confidence intervals, effect sizes, degrees of freedom and *P* value noted<br>*Give P values as exact values whenever suitable.*
☒	☐	For Bayesian analysis, information on the choice of priors and Markov chain Monte Carlo settings
☒	☐	For hierarchical and complex designs, identification of the appropriate level for tests and full reporting of outcomes
☒	☐	Estimates of effect sizes (e.g. Cohen's *d*, Pearson's *r*), indicating how they were calculated

Our web collection on statistics for biologists contains articles on many of the points above.

Software and code

Policy information about availability of computer code

Data collection	Agilent BioTek Gen5 Microplate Reader and Imager software (V.3.2), Thermo Scientific HCS Studio software (V.6.6.0), ZEN Imaging software (ZEN2 blue edition), LICOR Image Studio software (V.1.0.19), BD CellQuest Pro software (V.6.1), WOLFViewer software (V.2.4)
Data analysis	GraphPad Prism (V.9), FlowJo (V.10), Harmony (V.4.8), Adobe Photoshop (V.24.5.0), MODELLER (V.9.22), Chimera (V.1.14), PredUs (V.2.0), SPPIDER (V.2), consPPI (V.1.0), PINUP (V.1.0), ProMate (V.2), hierarchal clustering of competition binding ELISA and infection inhibition assays (GitHub: https://github.com/chandranlab/pcdh1_interface.git)

For manuscripts utilizing custom algorithms or software that are central to the research but not yet described in published literature, software must be made available to editors and reviewers. We strongly encourage code deposition in a community repository (e.g. GitHub). See the Nature Portfolio guidelines for submitting code & software for further information.

Data

Policy information about availability of data

All manuscripts must include a data availability statement. This statement should provide the following information, where applicable:

- Accession codes, unique identifiers, or web links for publicly available datasets
- A description of any restrictions on data availability
- For clinical datasets or third party data, please ensure that the statement adheres to our policy

The authors declare that the data supporting the findings of this study are available within the paper, its Supplementary Information files, and on GitHub (https://

github.com/chandranlab/pcdh1_interface.git). The data sets generated are available at Figshare, DOI: . The raw data generated in this study is provided in the Source Data file. The databases used in this study include PDB (6MGA) and GenBank (PCDH1 sequences, see above for accession numbers). Biological materials are available upon reasonable request from Rohit K. Jangra, Zhongde Wang, and Kartik Chandran.

Research involving human participants, their data, or biological material

Policy information about studies with human participants or human data. See also policy information about sex, gender (identity/presentation), and sexual orientation and race, ethnicity and racism.

Reporting on sex and gender	NA
Reporting on race, ethnicity, or other socially relevant groupings	NA
Population characteristics	NA
Recruitment	NA
Ethics oversight	NA

Note that full information on the approval of the study protocol must also be provided in the manuscript.

Field-specific reporting

Please select the one below that is the best fit for your research. If you are not sure, read the appropriate sections before making your selection.

☒ Life sciences ☐ Behavioural & social sciences ☐ Ecological, evolutionary & environmental sciences

For a reference copy of the document with all sections, see nature.com/documents/nr-reporting-summary-flat.pdf

Life sciences study design

All studies must disclose on these points even when the disclosure is negative.

Sample size	Sample sizes were chosen to obtain power > 0.80
Data exclusions	No independent experiments were excluded, however for some of the samples an individual well of one of the dilutions of one sample within one experiment was excluded. There was no pre-established criteria for exclusion, the wells that were excluded were determined aberrant based on the other dilutions of the same sample and technical replicates from all of the independent experiments.
Replication	To verify the reproducibility of the experimental findings, at least two technical replicates per experiment within a minimum of two independent experiments was performed for the cell culture rVSV infection experiments and ELISA binding experiments. The trends for the rVSV infection experiments were confirmed by authentic viruses by at least three technical replicates for a minimum of one independent experiment performed. As a control, wild-type sEC1-2 was performed with all binding experiments and all infection experiments on variant PCDH1 cell lines include the control wild-type PCDH1 cell line. For animal experiments, at least eight hamsters from each group were analyzed by ANDV challenge in one experiment. Two hamsters from each group were analyzed for PCDH1 expression in tissue samples in two independent experiments and three hamsters from each group (wild-type and double mutant) were analyzed for the presence of viral RNA and protein in one experiment. To protein gels for the the soluble protein was performed once and the verification of PCDH1 expression on cell lines was performed at minimum one experiment. The independent experiments that were replicated were reproduced.
Randomization	No randomization was used and covariates were not controlled as it was not applicable to this study as the variables between samples were of direct interest and did not contain extra variables that influenced the outcome.
Blinding	Data collection and analysis was not performed blind as the readouts and analysis of the data was performed the same way for all the samples and was not needed.

Reporting for specific materials, systems and methods

We require information from authors about some types of materials, experimental systems and methods used in many studies. Here, indicate whether each material, system or method listed is relevant to your study. If you are not sure if a list item applies to your research, read the appropriate section before selecting a response.

Materials & experimental systems

n/a	Involved in the study
☐	☒ Antibodies
☐	☒ Eukaryotic cell lines
☒	☐ Palaeontology and archaeology
☐	☒ Animals and other organisms
☒	☐ Clinical data
☒	☐ Dual use research of concern
☒	☐ Plants

Methods

n/a	Involved in the study
☒	☐ ChIP-seq
☐	☒ Flow cytometry
☒	☐ MRI-based neuroimaging

Antibodies

Antibodies used	PCDH1-specific antibodies include; monoclonal Ab (mAb) anti-EC1 "3305" and monoclonal Ab anti-EC7 "3677" from Donnelly Centre and Department of Molecular Genetics, University of Toronto, and polyclonal Ab (pAb) anti-PCDH1 catalog No. PA5-83876, ThermoFisher (RRID: AB_2791028). Anti-rabbit IgG Alexa Fluor™ 800 Plus pAb, catalog No. A32735, ThermoFisher. Anti-Flag clone M2 mAb, catalog No. F1804, Sigma-Aldrich (RRID: AB_262044). Anti-Flag clone M2-HRP-conjugate mAb, catalog No. A8592, Sigma-Aldrich (RRID: AB_439702). BetaActin mAb clone 8H10D10, catalog No. MA5-15452, ThermoFisher (RRID: AB_11001306). Anti-mouse IgG IRDye-800CW pAb, LI-COR. Anti-Human-IgG-HRP, pAb, catalog No. AP112, MilliporeSigma™. Anti-mouse-IgG-Alexa Fluor™ 488 pAb, catalog No. A-11001, ThermoFisher (RRID: AB_2534069). Anti-human-IgG-Alexa Fluor™ 488 pAb, catalog No. A-11013, ThermoFisher (RRID: AB_2534080), Anti-human-IgG-Alexa Fluor™ 555 pAb, catalog No. A-21433, ThermoFisher (RRID: AB_2535854). Anti-SNV N protein pAb #1244, USAMRIID. Anti-hamster-IgG-HRP, pAb catalog No. 5220-0371, SeraCare. Anti-ANDV N protein pAb, catalog No. NR-9673, BEI Resources. Anti-SNV N protein pAb, catalog No. NR-9674, BEI Resources. Anti-HTNV N protein pAb, catalog No.12152, BEI Resources.
Validation	Anti-EC1 "3305" and anti-EC7 "3677" were verified to bind to human PCDH1's EC1 and EC7 in previous experiments published by Jangra et al. (reference 6 in the manuscript) as well as in data provided in this manuscript. Anti-PCDH1 (PA5-838760), was verified to detect human PCDH1 by ThermoFisher for IF and IHC as well as by Western blot data in this manuscript. Mouse anti-Flag clone M2 mAb was verified by Sigma-Aldrich to detect Flag via Western blot, IF, and enzyme immunoassays. Mouse anti-Flag clone M2-HRP-conjugate mAb was verified by Sigma-Aldrich to detect Flag via Western blot and ELISAs. BetaActin mAb clone 8H10D10 was verified to target "beta-Actin in FACS, IF, and WB applications and shows reactivity with Hamster, Human, mouse, Non-human primate, and Rat samples." Anti-SNV pAb #1244 was verified by USAMRIID to detect SNV N protein via IHC. Anti-ANDV, SNV, and HTNV N protein pAbs were verified by USAMRIID in previous publication Jangra et al. (reference 6 in the paper) to detect ANDV, SNV, and HTNV N protein via IF.

Eukaryotic cell lines

Policy information about cell lines and Sex and Gender in Research

Cell line source(s)	Human osteosarcoma U2OS cells (ATCC) derived from a female osteosarcoma patient, Human umbilical vein endothelial cells (HUVEC, Lonza), Human pulmonary microvascular endothelial cells (HPMEC, Promocell), mouse primary lung microvascular endothelial cells (MLMEC, Cell Biologics), and Human FreestyleTM-293-F suspension cells (Thermo Fisher), Embryonic kidney fibroblast 293T cells (ATCC), grivet kidney Vero cells (ATCC), grivet kidney Vero E6 cells (ATCC), and Drosophila Schneider 2 cells (ThermoFisher/Gibco), stably expressing soluble ANDV GnH/Gc (Serris et al., 2020, reference 22 in the paper).
Authentication	HPMECs were authenticated by Promocell by cell morphology and cell-type specific markers, e.g. CD31 and Podoplanin using flow cytometry analyses. MLMECs were not authenticated. U2OS cells were authenticated by ATCC by Karyotyping and STR profiling. HUVECs were not authenticated. 293T cells were authenticated by ATCC by STR profiling. Vero cells were authenticated by ATCC by Karyotyping. Vero E6 cells were not authenticated. Drosophila Schneider 2 cells and FreestyleTM-293-F suspension cells were not authenticated.
Mycoplasma contamination	All cell lines were subjected to routine mycoplasma testing and were found to be negative.
Commonly misidentified lines (See ICLAC register)	No misidentified cell lines were used in this study.

Animals and other research organisms

Policy information about studies involving animals; ARRIVE guidelines recommended for reporting animal research, and Sex and Gender in Research

Laboratory animals	Wild-type, PCDH(F83A/D85R), and PCDH1(10 a.a.) Syrian golden hamsters (Mesocricetus auratus), male and Female, 5-12 weeks old, were used in this study.
Wild animals	No wild animals were used in this study.
Reporting on sex	Male and female animals were both used and the findings did not apply to only one sex. Sex-based analysis was not performed as large differences in survival between males and females in lethal ANDV challenge has previously not been observed.

Field-collected samples: No field collected samples were used in this study.

Ethics oversight: The studies were conducted under IACUC-approved protocols in compliance with the Animal Welfare Act, PHS Policy. The facilities where the study was conducted (Utah State University and USAMRIID) are accredited by the Association for Assessment and Accreditation of Laboratory Animal Care, International (AAALAC), and adhere to principles stated in the Guide for the Care and Use of Laboratory Animals, National Research Council, 2011. USAMRIID IACUC approved the protocols for the studies conducted at USAMRIID and Utah State University IACUC approved the protocols for the studies conducted at Utah State University.

Note that full information on the approval of the study protocol must also be provided in the manuscript.

Flow Cytometry

Plots

Confirm that:

☒ The axis labels state the marker and fluorochrome used (e.g. CD4-FITC).

☒ The axis scales are clearly visible. Include numbers along axes only for bottom left plot of group (a 'group' is an analysis of identical markers).

☒ All plots are contour plots with outliers or pseudocolor plots.

☒ A numerical value for number of cells or percentage (with statistics) is provided.

Methodology

Sample preparation: Live human U2OS cells expressing variant PCDH1 were kept on ice and blocked with PBS/10% FBS at 4°C. Surface PCDH1 was stained using a human anti-EC7 mAb-3677 (5 µg/mL) followed by an anti-human IgG Alexa Fluor™ 488 (1:500 dilution, ThermoFisher). After washing, cells were re-suspended in PBS/2% FBS or PBS/3%BSA and passed through a 0.41 µm Nylon Net Filter (Millipore). For cells that were sorted, TO-PRO-3 Ready Flow Reagent (Invitrogen) was added before sorting to distinguish live/dead cells.

Instrument: LSRII Flow Cytometer (BD Biosciences) and WOLF G2 Cell Sorter (NanoCellect) were used for data collection.

Software: CellQuest Pro software (V.6.1) and WOLFViewer software (V.2.4) were used to collect and FlowJo 10.8.1 software was used to analyze the flow cytometry data.

Cell population abundance: U2OS cells expressing mutant PCDH1 had an abundance of ~95-98% PCDH1+ cells out of the total cells analyzed. For the cells that were sorted, cells expressing high levels of PCDH1 constituted around 60% for the cells expressing wild type PCDH1, and ~25% of cells expressing a mutant version (10 a.a.) PCDH1. Post-sort, the abundance was similar between the two cell lines ~95%. Purity was determined by flow cytometry and immunofluorescence microscopy, comparing PCDH1 expression between the two cell lines relative to U2OS-PCDH1-KO cells.

Gating strategy: The gating strategy included an initial gate to exclude cell debris, gating on cells using forward scatter height (FSC-H) and side scatter height (SSC-H) parameters or FSC-H and Back-scatter height (BSC-H). For sorting experiments, an additional gate was used to select for live cells (those negative for far-red, 7AAD). Cells were gated on their PCDH1 expression (FIT-C) using PCDH1-KO cells as "negative" and cells expressing wild type PCDH1 as "positive". Sorted cells were then further gated based on the level of PCDH1 expression (high or low) determined by a strong peak for high level expressing cells exhibited by those cells expressing wild type PCDH1.

☒ Tick this box to confirm that a figure exemplifying the gating strategy is provided in the Supplementary Information.
