# Supplementary material for: Two point mutations in protocadherin-1 disrupt hantavirus recognition and afford protection against lethal infection
Source: Nat Commun. 2023 Jul 24;14:4454. doi: 10.1038/s41467-023-40126-y (PMC10366084; doi:10.1038/s41467-023-40126-y)
Supplement: Supplementary file 4 — Description of Additional Supplementary Files [file 41467_2023_40126_MOESM4_ESM.pdf]

Supplementary Data 1
Alignment of PCDH1 and EC1 amino acid sequences with a selection of rodent and primate species. The residues within EC1 which deviate from human EC1 are in bold, red font.

Supplementary Data 2
Interfacial predictions from five different methods on the residues within PCDH1's EC1-4. The first tab includes the interfacial prediction summary for each residue, and the second tab includes the individual interfacial prediction for each residue and given conformation (open/close) and the union (open+close).
